# Identifying typical physical activity on smartphone with varying positions and orientations

**DOI:** 10.1186/s12938-015-0026-4

**Published:** 2015-04-13

**Authors:** Fen Miao, Yi He, Jinlei Liu, Ye Li, Idowu Ayoola

**Affiliations:** The Key Lab for Health Informatics of Chinese Academy of Sciences, Shenzhen Institutes of Advanced Technology, Chinese Academy of Sciences, Shenzhen, China; Shenzhen College of Advanced Technology, University of Chinese Academy of Sciences, Beijing, China; High-field Magnetic Resonance Department, Max Planck Institute for Biological Cybernetics, Tuebingen, Germany; School of Electrical & Mechanical Engineering, Guangdong University of Technology, Guangzhou, China; Industrial Design, Technische Universiteit Eindhoven, 5612 Eindhoven, AZ the Netherlands

## Abstract

**Background:**

Traditional activity recognition solutions are not widely applicable due to a high cost and inconvenience to use with numerous sensors. This paper aims to automatically recognize physical activity with the help of the built-in sensors of the widespread smartphone without any limitation of firm attachment to the human body.

**Methods:**

By introducing a method to judge whether the phone is in a pocket, we investigated the data collected from six positions of seven subjects, chose five signals that are insensitive to orientation for activity classification. Decision trees (J48), Naive Bayes and Sequential minimal optimization (SMO) were employed to recognize five activities: static, walking, running, walking upstairs and walking downstairs.

**Results:**

The experimental results based on 8,097 activity data demonstrated that the J48 classifier produced the best performance with an average recognition accuracy of 89.6% during the three classifiers, and thus would serve as the optimal online classifier.

**Conclusions:**

The utilization of the built-in sensors of the smartphone to recognize typical physical activities without any limitation of firm attachment is feasible.

## Background

Physical activity is becoming a widespread public concern due to its extensive health benefits, especially on reducing risk of chronic diseases [1]. Daily activities information is useful for clinicians to accurately diagnose chronic diseases [2] by providing contextual information while analyzing vital-signs of patients over a period of time. The physical activity recognition technique can not only be used to promote the users to increase physical activity [3], but also can improve the differentiated treatment for the clinicians on diagnosis of neurological, degenerative and respiratory disorders [4,5]. Mobile activity monitoring also provides us with an opportunity for social interaction whenever and wherever possible [6].

Although many activity recognition approaches [7,8] have been developed with good performance, they have difficulties in widely application due to high cost and inconvenience to use with so many sensors around human body. Wearable sensors have been proved in previous studies for its feasibility and effectiveness in activity recognition [9-12], but sometime users may forget to wear the clothes with micro sensors. Smartphone are now deployed with a variety of built-in sensors for many other features in addition to the basic telephony, which can be used to monitor activity. It was also demonstrated in [13] that gait parameters acquired from smartphone are with a degree of accuracy comparable to that of the tri-axial accelerometer [13]. Until now, several physical activity recognition methods have been proposed using smartphone sensors. In 2012, Cho et al. proposed a linear discriminant analysis based activity recognition method using support vector machine to classify five activities including walking, going upstairs, going downstairs, running and static [14]. Anjum et al. evaluated 4 machine learning algorithms including Naiive Bayes, Decision tree, K-Nearest Neighbour and Support Vector Machine classifiers to recognize seven activities (walking, running, climbing/descending stairs, driving, cycling, and inactive) using smartphone accelerometer and gyroscope [15]. The decision tree was demonstrated to be the best classifier with the average AUC (area under the ROC curve) larger than 0.9. Arif et al. proposed to track physical activities with acceleration sensor incorporated in the smartphone in front pants leg pockets [16]. Six activities were recognized based on the extracted 105 features and then reduced to 30 using K-Nearest Neighbour classifier. To make the activity recognition solution more flexible, a phone-position independent algorithm was developed in [17] to recognize seven activities based on a complex method to analyze movement periodicity. In addition, some unsupervised learning algorithms were used for human activity recognition to avoid generating a large number of labelled activities for the training dataset [18-20]. Compared with supervised learning method, unsupervised algorithms are often weak in accuracy and little number of activities recognized.

In built-in sensors of smartphone, three of them are hardware-based (the accelerometer, gyroscope and magnetic sensor), while others can be either hardware-based or software-based (the gravity, linear acceleration, and rotation vector sensors). Moreover, the software-based sensors derive their data from the accelerometer, magnetic sensor or gyroscope. The software-based sensors are more varying because they often rely on one or more hardware sensors. Therefore, hardware-based sensors are chosen for monitoring movements in our study, such as tilt, shake, rotation, or swing. In another aspect, different from previous studies [21,22], in which the smartphones were fixed in a certain position and orientation of the body and thus would restrict normal behaviours while using the device, we will dedicate to a flexible activity recognition solution with the phone in our pocket or bag, which is closer to the users’ habits. It was also demonstrated that when we placed a mobile phone in our pocket or bag, it moved with the pace of our body, thus it appeared to be an ideal location to detect the activities of the user [23].

In our study, the smartphone is freely placed in a user-determined pocket and we choose five most representative daily activities that are strongly linked to physical exercises for classification. The main purpose of this paper is to automatically detect whether the smartphone is worn in the pocket before activating physical activity recognition without any limitation of firm attachment. After judging whether the phone is in a pocket, thirty statistical features were extracted from five signals generated by the accelerometer, gyroscope and magnetic sensor that are insensitive to orientation for classification. Decision trees (J48), Naive Bayes and Sequential minimal optimization (SMO) in the WEKA Machine Learning Toolkit [24] were employed to recognize five activities: static, walking, running, walking upstairs and walking downstairs. The average recognition accuracy of 89.6% with J48 demonstrated the feasibility of the proposed solution. We also investigated the influence of positioning the smartphone in six pockets with 4 orientations to demonstrate the flexibility of the proposed solution.

## Materials and methods

**Figure 1:**
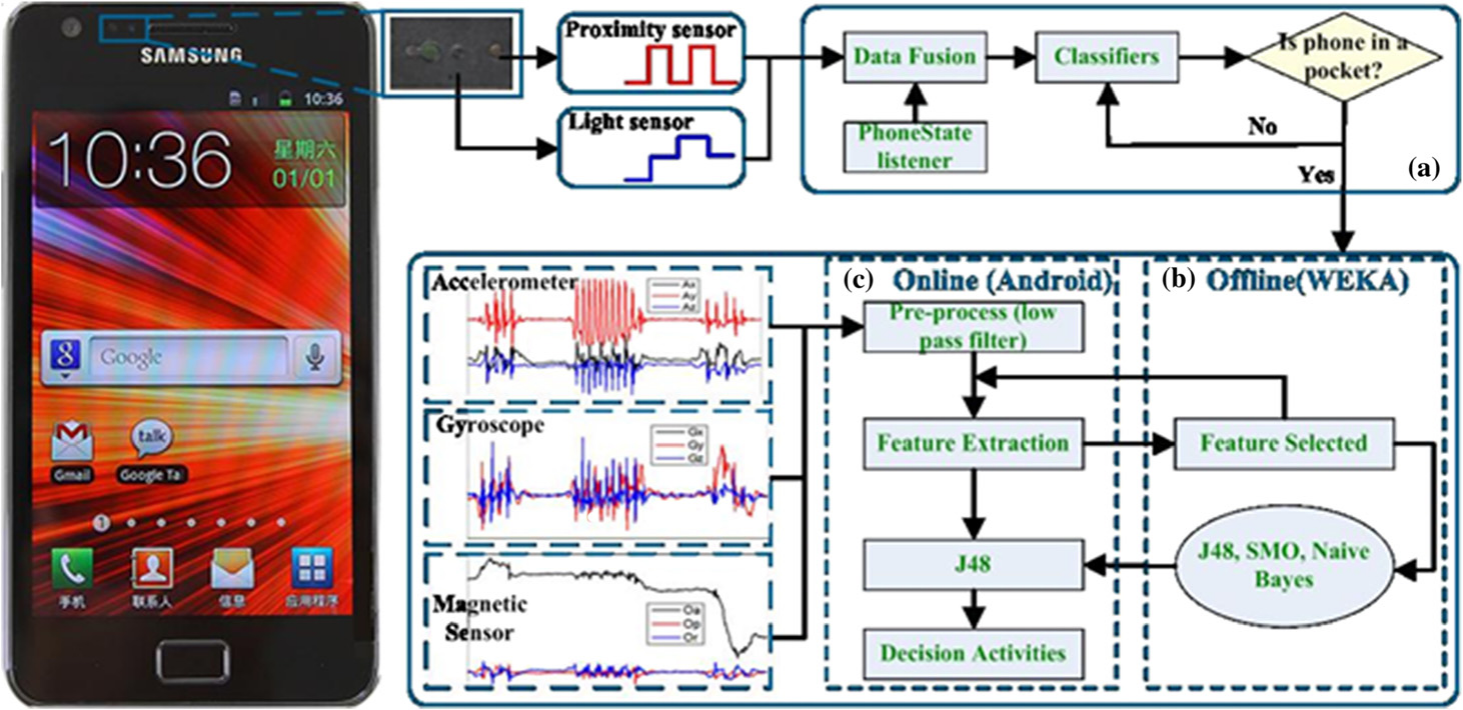
Block diagram of the recognition scheme. **(a)** The part to determine whether the phone is in a pocket. **(b)** In WEKA environment offline activities classification **(c)** In real-time online activities classification.

The built-in accelerometer, gyroscope, proximity sensor, light sensor and the magnetic sensor of a smartphone was used to collect information that reflected acceleration, angular velocity, distance, light changes and orientation of physical activities. Signals were extracted from the data and the optimal signals were selected in order to classify activities. Due to the loose placement of the smartphone with varying orientations, we aim to extract signals that are independent or insensitive to orientation for the activity classification. The block diagram of the proposed recognition scheme of this work is illustrated in Figure 1.

### Device and study population

**Figure 2:**
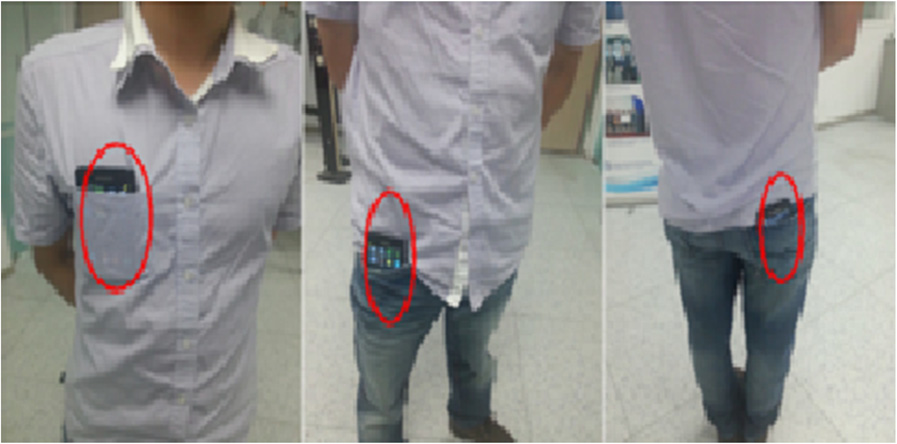
Pocket locations. For each pocket shown, there is a corresponding one in the left side of the body.

A smartphone (Samsung, I9100GALAXYSII, 125.3 × 66.1 × 8.49 mm3, 116 g, Android OS 2.3) was worn on six body positions without affixing it, the positions were the two front and back pockets on the trousers and the two front pockets on the coat, as shown in Figure 2. The smartphone has a built-in proximity sensor, a light sensor, a triaxial accelerometer (STM K3DH) with 19.6 m/s2 resolution, a triaxial gyroscope sensor (STM K3G) with 34.9 rad/s maximum range and 0.0012 rad/s resolution, and a triaxial magnetic field sensor (Asahi Kasei AK8973) with 2000 μT maximum range and 0.0625 μT resolution.

Seven healthy volunteers (4 males and 3 females) were recruited to generate a representative dataset for activity recognition. The baseline characteristics of the subjects were: age 30 ± 5 years (range 25–36), body weight 65 ± 20 kg (range 44–83), and body mass index 22.0 ± 2.8 kg/m2 (range 18.2-25). Every subject was informed and provided informed consent before the experiment.

### Identify the location of mobile phone

**Figure 3:**
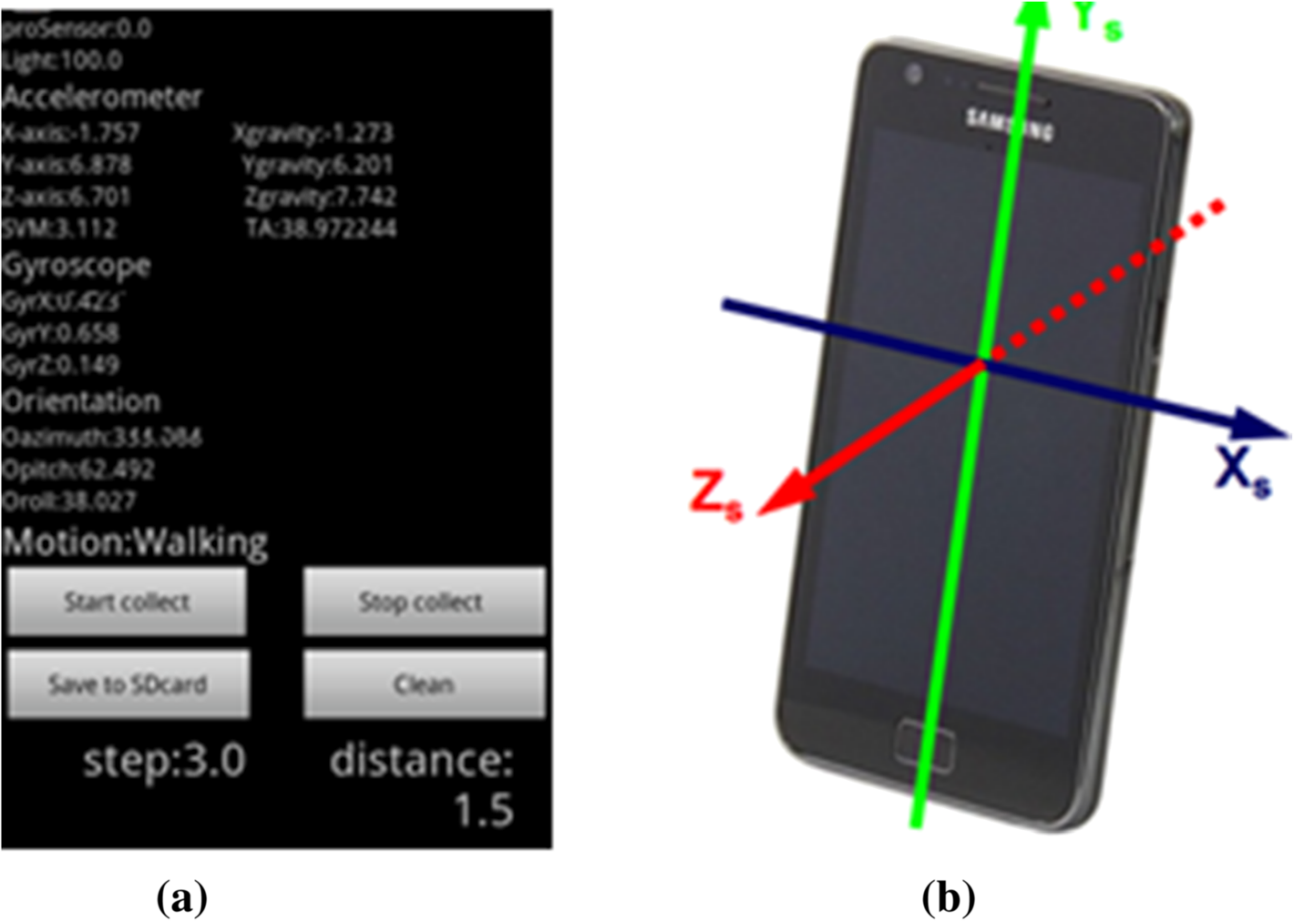
Software interface and coordinate system of the smartphone. (**a**) Data collection interface on Samsung I9100. (**b**) The coordinate system of the smartphone. For each pocket shown, there is a corresponding one in the left side of the body.

As our study aims to recognize human activity based on the smartphone in the user’s pocket, we will automatically identify whether the mobile phone is in the user’s pocket before analysis with the help of proximity and light sensors. It was also presented in [25] that the proximity and light sensors of the phone could realize simple forms of context recognition associated with the user interface. Information about light and proximity sensors is shown in Table 1. The proximity sensor can determine how far an object is from a device. It is usually used to determine how far a person’s head is from the front of a handset, for example, when a user is making or receiving a phone call. Most proximity sensors return the absolute distance, in cm, but some return only near and far values. Therefore, we will use the light and proximity sensors to determine whether the phone is in the user’s pockets. Take Samsung I9100 for example, if an object is within a close range or out of range, it will read 0.0 or 5.0 values respectively (see Figure 3(a)). The light sensor measures the ambient light level in lux. The android platform supported eight different luminance values, as shown in Table 2.

**Table 1:** **Description of light sensor and proximity sensor**

**Sensor**	**Type**	**Description**	**Common uses**
LIGHT	Hardware	Measures the ambient light level (illumination) in lux.	Controlling screen brightness.
PROXIMITY	Hardware	Measures the proximity of an object in cm relative to the view screen of a device. This sensor is used to determine whether a handset is being held up to a person’s ear.	Phone position during a call.

**Table 2:** **Eight luminance values supported by Android platform**

**Type**	**Description**	**Constant value (lux)**
LIGHT_NO_MOON	luminance at night with no moon in lux	0.0010
LIGHT_FULLMOON	luminance at night with full moon in lux	0.25
LIGHT_CLOUDY	luminance under a cloudy sky in lux	100.0
LIGHT_SUNRISE	luminance at sunrise in lux	400.0
LIGHT_OVERCAST	luminance under an overcast sky in lux	10000.0
LIGHT_SHADE	luminance in shade in lux	20000.0
LIGHT_SUNLIGHT	luminance of sunlight in lux	110000.0
LIGHT_SUNLIGHT_MAX	Maximum luminance of sunlight in lux	120000.0

From Tables 1 and 2 we can see, the phone can be considered as in a pocket when the luminance value is less than 100 lux and the proximity sensor returns 0.0. But there is a special case that the user is making or receiving a phone call at night. In order to judge the special case, we register a Phone State Listener event to monitor the phone state change in our application. The performance of identifying the locations of the smartphone using light and proximity sensors and Phone State Listener event will be given in the Results section.

### Data collection

An application software of physical activity management was developed and installed on the investigator’s smartphone that measure distance, intensity of light, acceleration, angular velocity and orientation, as shown in Figure 3(a). As shown in Figure 3(b), the standard sensor coordinate system of the smartphone is defined relative to the screen. The X-axis is horizontal and points to the right, the Y-axis is vertical and points up, and the Z-axis points toward the outside of the screen. The coordinate system of the accelerometer is the same as the standard sensor coordinate system, so the acceleration of A_x_, A_y_, A_z_, show the accelerometer of X, Y, Z direction, respectively. The software-based low pass filter with 0.25 Hz cutoff frequency was employed to separate acceleration due to gravity (GA) and linear acceleration (LA). So A_x_ was separated to GA_x_, and LA_x_, the same for A_y_ and A_z_ . And the coordinate system of the gyroscope is the same as the accelerometer. The raw acceleration, gyroscope and orientation signals were sampled at 25 Hz and stored in text format on Secure Digital (SD) card in the smartphone and then transferred to computer for further analysis.

In order to facilitate data collection, the light and proximity sensors were used to automatically control the data acquisition. Once the phone was put into subject’s pocket, the data collection would start working, if it was taken out it would stop collecting data.

In order to reflect the real living status, the experimental protocol was designed as flexible as possible that each subject was asked to perform each activity listed in Table 3 in their own style and in a random order with each activity lasting for 1 minute each time with the smartphone in one pocket. Six pocket positions presented in Figure 2 will be investigated in our study. In order to mark the data efficiently, the subject was kept stationary for 5 seconds between two different activities. The data at the first and last 5 seconds were cut off to stable the signal since subjects need time to put the phone inside the pocket and also take it out. Almost 300 activity data were derived from 5-minute motion with a resolution of 0.8 s in one pocket for each subject. With the proposed 6 pocket position, nearly 1800 activity data were derived from one subject. As we aim to recognize activities independent of subjects, all the activity data collected from the seven subjects’ six pocket positions were mingled together to establish an independent dataset. Overall 8097 data were collected with 1697, 2531, 2080, 675 and 1114 for the 5 activities listed in Table 3 separately.

**Table 3:** **Activities performed in this experiment**

**Number**	**Activity task**	**Activity description**
**1**	Static	Standing still/sitting on a sofa/sitting at a desk
**2**	Walking	Walking on a treadmill/walking on the playground
**3**	Running	Running on a treadmill/running on the playground
**4**	Walking downstairs	Walking downstairs at a normal pace
**5**	Walking upstairs	Walking upstairs at a normal pace

The original physical activity data, accompanied with the corresponding multiple features extracted from the sensors of the smartphone, are freely available at https://github.com/fenmiao/ActivityData for further study.

### Feature extraction

**Figure 4:**
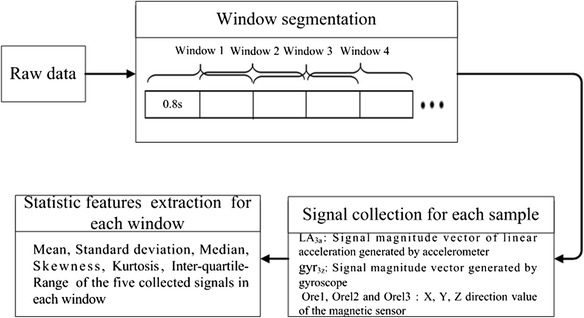
Feature extraction process.

The proposed feature extraction process can be expressed as Figure 4. Before feature extraction, to reduce bias due to sensor sensitivity and noise, sliding window approach with 50% overlap was employed to divide the signal into smaller time windows with each window of 1.6 seconds.

Different from most previous works, which realize activity recognition using body-worn sensors that are fixed on a specified body location and orientation, the orientation and position of the smartphone in our study can be varying, according to the users’ actual status. We chose two magnitude signals that are insensitive to orientation and position, LA_3a_ and gyr_3z_ for classification. LA_3a_ is the signal magnitude vector (SMV) of linear acceleration, which can be represented as:

1 $$ L{A}_{3a}=\sqrt{L{A}_x^2+L{A}_y^2+L{A}_z^2} $$

Likewise, gyr_3z_ uses the same computational method as LA_3a_ with the gyroscope sensor.

In another aspect, Ore1, Orel2 and Orel3, which represents the X, Y, Z direction value of the magnetic sensor, were collected as the 3 independent signals. Combined with LA_3a_ and gyr_3z_, 5 signals were collected in our study.

The above signals were extracted from each sliding window and then for statistical processing. Six kinds of statistical features for all the windows with high popularity in many pattern recognition and machine learning problems were computed for activity classification. They are defined as follows:

- Mean: the average value of the signal over the window
- Standard Deviation: the standard deviation value over the window
- Median: the median value over the window
- Skewness: the statistic value to describe the overall distribution of all values in the form of steep slow degree,
  2 $$ \begin{array}{l} Skewness=\frac{n}{\left(n-1\right)\left(n-2\right)}{\displaystyle \sum_{i=1}^n{\left({x}_i-Avg\right)}^3/st{d}^3}\\ {}\end{array} $$
  Where Avg is the mean of x_i_, std is the standard deviation of x_i_.
- Kurtosis: the degree of peakedness of the distribution over the time window,
  3 $$ Kurtosis=\frac{n\left(n+1\right){\displaystyle \sum {\left({x}_i-Avg\right)}^4-3{\left({{\displaystyle \sum \left({x}_i-Avg\right)}}^2\right)}^2\left(n-1\right)}}{\left(n-1\right)\left(n-2\right)\left(n-3\right)st{d}^4} $$
  Where Avg is the mean of x_i_, std is the standard deviation of x_i_.
- Inter-quartile-Range (IR):
  4 $$ IR={Q}_3-{Q}_1 $$
  Where Q_3_, Q_1_ is the 75th and 25th percentiles over the window, respectively.
  At last, a 30 feature vector was obtained for classification, which was also presented in Figure 4.

### Classification

**Figure 5:**
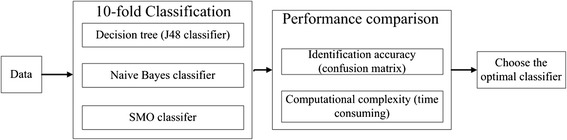
Data classification process.

We classified the five activities from daily life data presented above employing three different classifiers in WEKA environment [24]. The data classification process is presented in Figure 5. We compared and evaluated the performance of three activity recognition classifiers: decision trees (J48), Naive Bayes and Sequential minimal optimization (SMO). In order to give insights on how the models will generalize to an independent dataset with large number of features (overall 30 features) and relatively small size of data, cross validation [26] were employed. In k-fold cross-validation, the original sample is randomly partitioned into k equal size subsamples. A single subsample is selected from the k subsamples as the validation dataset for testing the model, and the remaining k-1 subsamples are used as the training dataset. The cross-validation process repeat for k times, with each of the k subsamples used exactly once as the validation data. The results from the k processes can then be averaged to produce a single estimation. In general, 10-fold cross-validation is commonly used in most of situations. Therefore, 10-fold cross validation was used to optimize the three classifiers in our study.

1. J48 is an open source Java implementation of the C4.5 algorithm in the weka data mining tool. It builds decision trees from the training dataset based on the concept of information entropy. The following design elements should be considered in the training phase:
  At each node of the tree, the attribute of the data that splits its sample set into subsets concentrated in one class or the other most effectively, that is, with the highest normalized information gain, is chosen to make a decision.
  A stop-splitting rule is required to control the growth of the tree, and the terminal node is declared as a leaf node.
  If a smaller tree structure was able to achieve a performance comparable to a larger one, the smaller one was chosen. It should be noted that the size of the tree might be different in each training phase.
  J48 was chosen to give results of the decision tree, which can be easily transformed for real-time applications. And it has been successfully applied to activity recognition earlier. The parameters for the J48 decision tree are defined as follows:
  (i) confidence factor = 0.25;
  (ii) minimum number of objects = 30, numFolds = 3;
  (iii) unpruned = True.
2. In the Naive Bayes [27] classification scheme, the estimate of the probability density functions (PDF) at a point *x* = [*x*(1), …, *x*(*l*)]^*T*^ ∈ *ℜ*^*l*^ is given as
  5 $$ p(x)={\displaystyle \prod_{j=1}^lp\left(x(j)\right)} $$
  That is, the components of the feature vector x are assumed to be statistically independent. In order to ensure good estimates of the PDF, the number of the training samples, N, must be large enough. Although the independence assumption may not be valid with the Naive Bayes classifier, the final result turns out to be that the Naive Bayes classifier can be very robust. And it has been reported to perform well for many real-world data sets.
3. Instead of previous SVM learning algorithms that use numerical quadratic programming (QP) as an inner loop, SMO uses an analytic QP step by decomposing the overall QP problem into a series of small QP problems. SMO chooses to solve the smallest possible optimization problem at every step. For the standard SVM QP problem, the smallest possible optimization problem involves two Lagrange multipliers, because the Lagrange multipliers must obey linear equality constraint. At every step, SMO chooses two Lagrange multipliers to jointly optimize, find the optimal values for these multipliers, and updates the SVM to reflect the new optimal values. The advantage of SMO is that solving for two Lagrange multipliers can be done analytically. Thus, numerical QP optimization can be avoided entirely. The inner loop of the algorithm can be expressed in a short amount of C code, rather than invoking an entire QP library routine. Even though more optimization sub-problems are solved in the course of the algorithm so that each sub-problem is so fast that the overall QP problem can be solved quickly [28], the operation is time-consuming while the training data set is very large. So we just employ this method for comparison.
  Classification performances for above three classifiers were measured by confusion matrix, which will be presented in the Results section.

## Results

### The result of recognizing whether the phone is in the pocket

In our study, motion sensors start to collect data only when the phone has been put inside the pocket. In order to judge whether the phone is put inside the pocket or not, we set the following rules:

1. The proximity sensor: If the proximity sensor returns near, we use the value 0 to indicate the near state, otherwise we use the value 1 to indicate the far state.
2. The light sensor: Normalize the intensity value of light sensor of Table 3 to the range [0, 1].
3. The Listener for Call State: The Android system provides LISTEN_CALL_STATE in the class of PhoneStateListener which is the listener for monitoring changes in call state on the device. When the phone is calling, the value 1 is used to indicate the call state, otherwise the value 0 is utilized to indicate the uncalled state.

**Figure 6:**
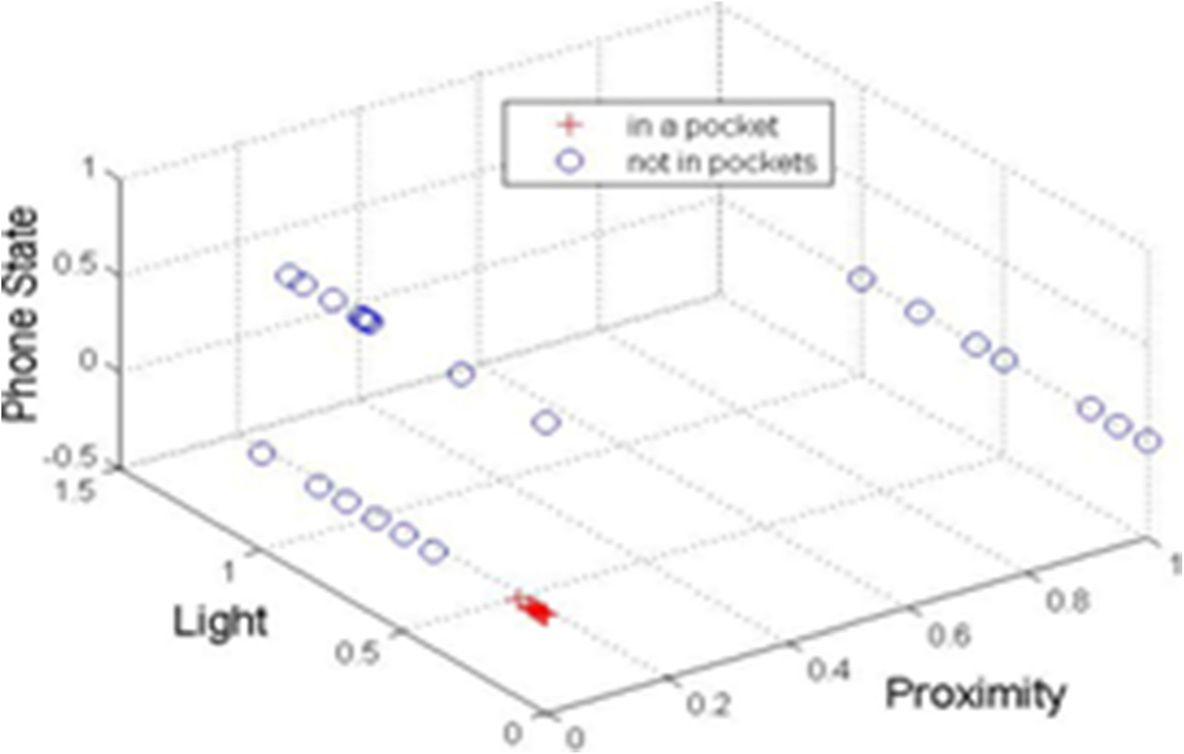
Classification result of the phone’s position.

From the classification result demonstrated in Figure 6, we can see the in-pocket case can be well identified according to the proposed rules in our study.

### The activities recognition results

**Figure 7:**
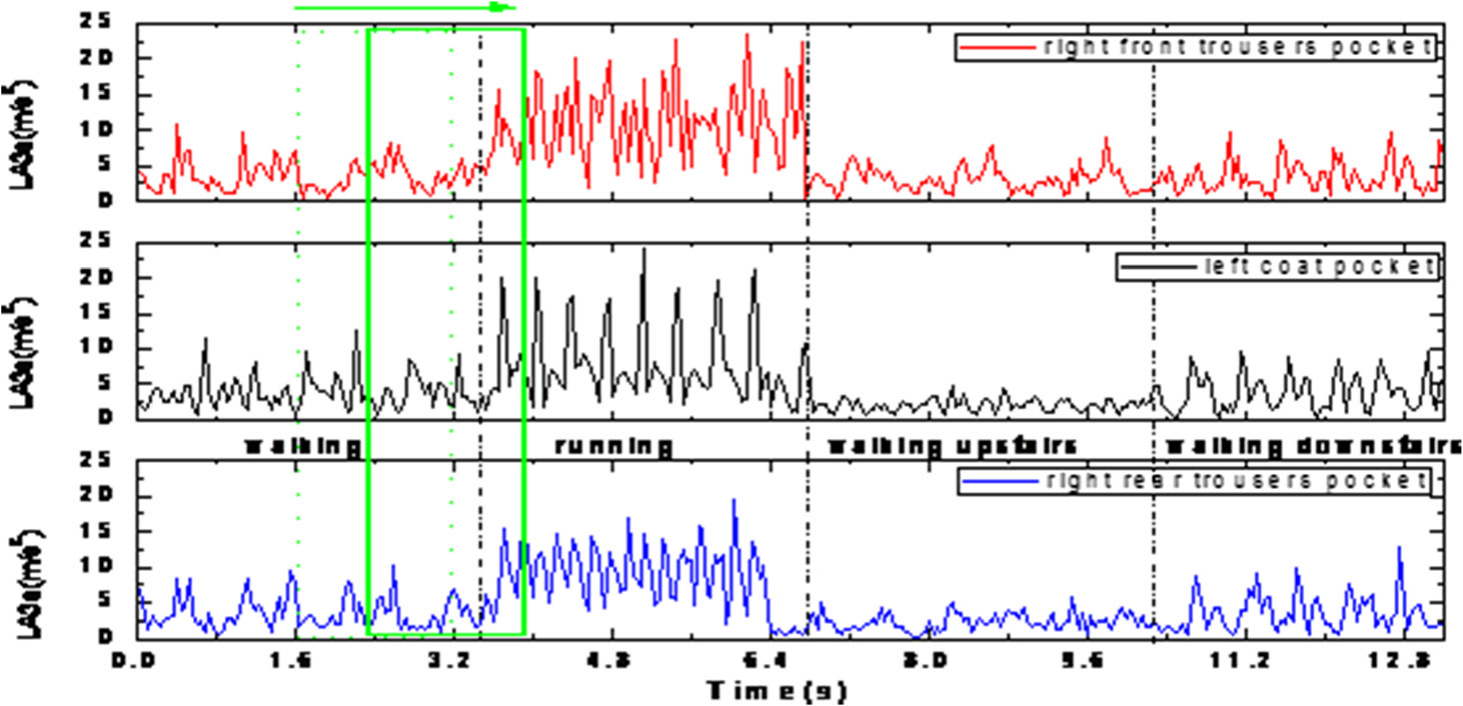
Acceleration data collected from three different positions.

As the features were extracted from the sliding windows, classification was done with 0.8 seconds time resolution. An example of one subject’s LA_3a_ curves from three different positions is shown in Figure 7. From the figure we can see, the value of LA_3a_ in coat pocket is smaller than that in trousers pockets as bigger acceleration would be produced from the lower limbs than that from the trunk part while the subject is moving. In another aspect, the waveform of the coat pocket seems to be more regular as the phone in trousers pockets often shakes more severely than that in coat pockets.

**Figure 8:**
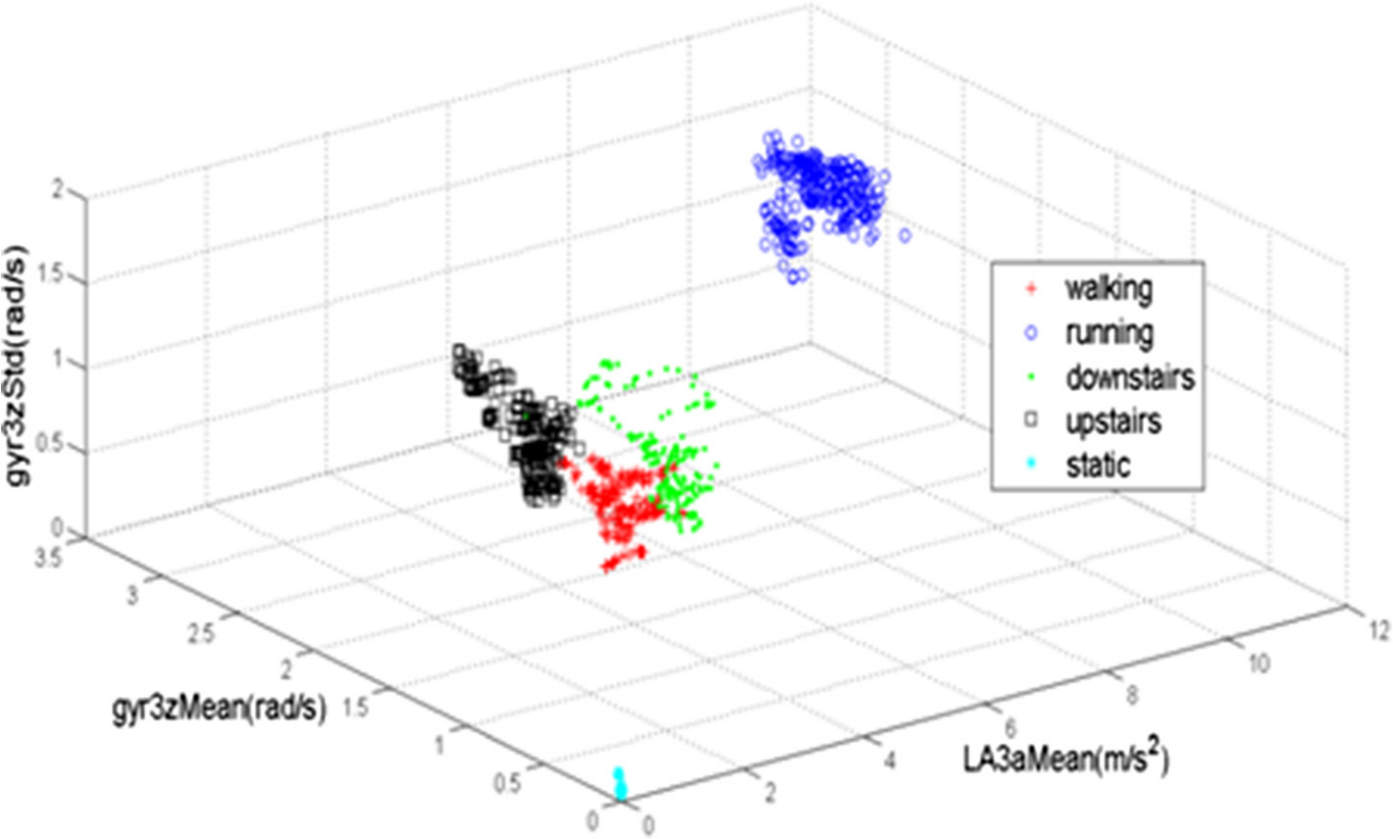
The scatter graphs of five activities, employing a combination of gyr3zStd, gyr3zMean and LA3aMean.

The scatterplot of five activities employing a combination of gyr3zStd, gyr3zMean and LA3aMean are shown in Figure 8. In Figure 8, the star-shape scatter area represents static, the plus sign scatter area represents walking, the circle scatter area represents running, the diamond scatter area represents downstairs and the square scatter area represents upstairs. From Figure 8 we can see, the distribution of data from the five activities are with a certain degree of overlap with just gyr3zStd, gyr3zMean and LA3aMean, especially for waking, walking upstairs and walking downstairs. Two reasons may be responsible for this phenomenon. Firstly, the subject needs time to switch from one activity to another but the reading of sensors is consecutive. The features of the switch time maybe confused between the two adjacent activities. Secondly, the same acceleration magnitude will be produced while subject is walking upstairs or downstairs and this will result in visible recognition inaccuracy.

Table 4 shows the confusion matrix of the J48 classifier developed based on the proposed 30 statistical features, with each row representing the number of activity data classified to one class with the proposed model and each column showing the actual number of activity data belonging to all classes. The performance of the three classifiers in terms of average recognition accuracy and time taken to build the model on recognizing five activities is listed in Table 5. From the table we can see, decision tree classifiers showed the best performance with an overall accuracy of 89.6% for the five activities. Therefore, in the real application of physical activity recognition, the J48 was chosen as the optimal classifier to recognize activities real-time with the highest accuracy and reasonable time to build the model.

**Table 4:** **Confusion matrix of J48 decision tree**

**Model actual**	**Walking upstairs**	**Walking downstairs**	**Walking**	**Running**	**Static**
**Walking upstairs**	*915*	18	160	21	0
**Walking downstairs**	22	*454*	158	41	0
**Walking**	127	85	*2268*	51	1
**Running**	23	51	68	*1938*	0
**Static**	0	0	16	0	*1681*

**Table 5:** **Classification results**

**Methods**	**Accuracy (%)**	**Root mean squared error**	**Time taken to build model (s)**
**J48**	89.6	0.1804	0.65
**Naive Bayes**	75.3	0.283	0.12
**SMO**	81.1	0.332	1.74

## Discussion

Most previous studies [15-22,29-31] have focused on recognizing activities with wearable sensors. However, most of these works required accelerometers to be attached to a specific location. Table 6 gives the comparison between the previous typical studies with the proposed method in our study. From the table we can see, the proposed solution in our study can automatically identify the locations of the smartphone and realize convenient activity recognition from the smartphone at any pockets with quite good accuracy. Even though in-pocket case in this paper is an ideal position to realize quite high accuracy, we believe that an efficient solution in the future study to deal with more cases, such as in the hand and making a call, is needed to adapt to more situations.

**Table 6:** **Comparison with the reported activity recognition methods**

**Reference**	**Smartphone position**	**Activities numbers**	**Contributions**	**Algorithm and accuracy**	**Limitations**
Anjum et al. [15], 2013	Pant pocket, hand, hand bag, shirt pocket	7	Activity recognition with smartphone at multiple positions including pant pocket, hand, hand bag and shirt pocket	Decision tree (AUC 0.985)	Limited activity traces and thus would tradeoff the performance in external verification
Arif [16], 2014	Leg front pants pocket	6	Demonstration of better activity classification accuracy	10-fold KNN (98.2%)	Position is fixed in front pants leg pockets
Romain Guidoux et al. [17], 2013	Leg front pants pocket	9	Estimation of total energy expenditure with phone-position independent by transform	Total energy expenditure (73.6%)	Low accuracy
Yongjin Kwon et al. [19], 2014	Pants pocket	5	Unsupervised learning without labels	Hierarchical clustering or DBSCAN (above 90% accuracy)	Some important activities including going upstairs and downstairs were not studied
Sourav Bhattacharya [20], 2014	Jacket pockets, pants pockets, backpack	8	Deal with unlabeled data	Sparse coding (80%)	Important activities including going upstairs and downstairs were not studied
This paper	Any pockets	5	Automatically identify the locations of the smartphone and conveniently activity recognition with smartphone at any pockets	10-fold J48 (89.6%)	More situations, such as in the hand, should be further studied

**Figure 9:**
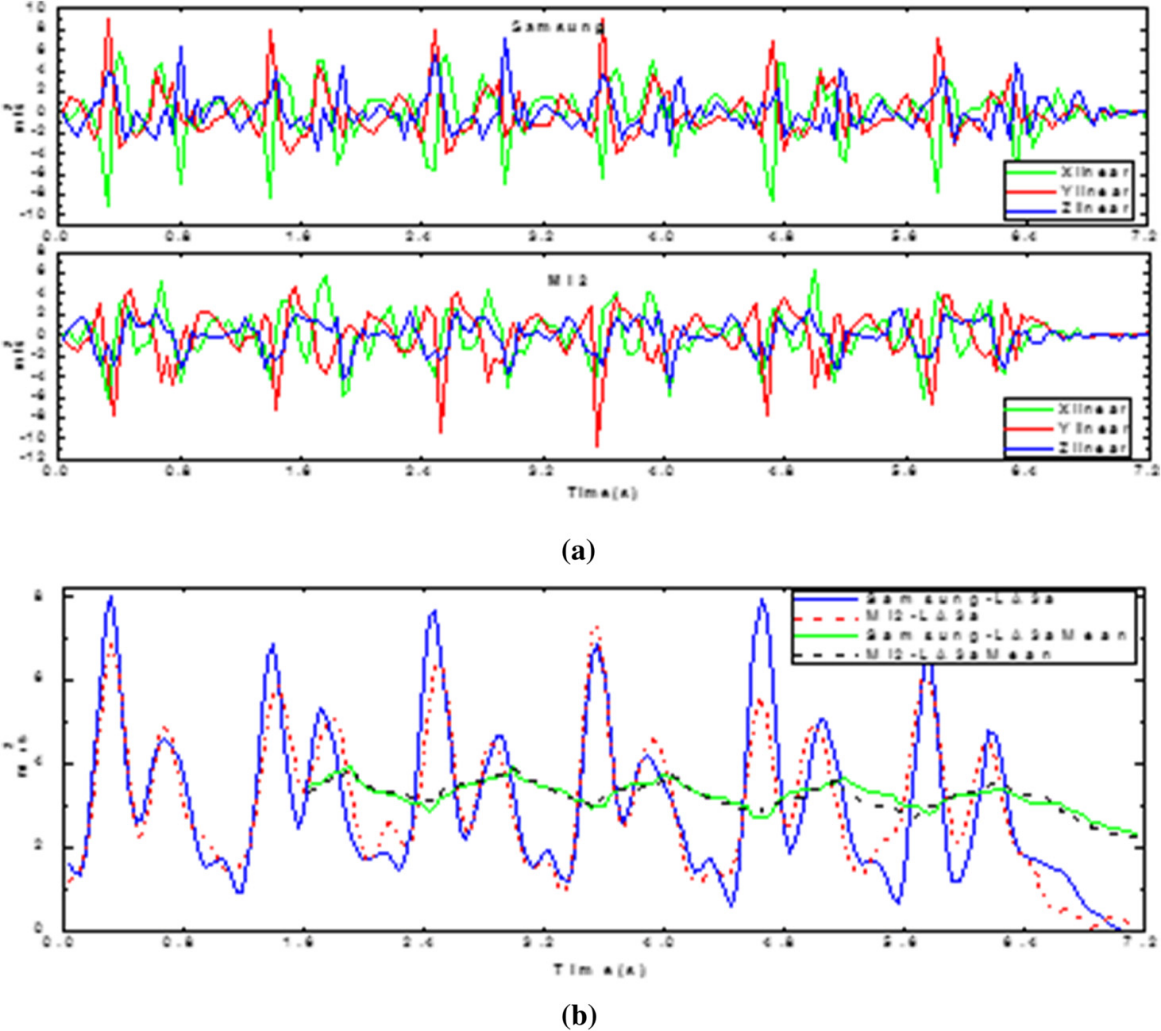
Acceleration data collected from four different orientations of the smartphone. **(a)** The three axis linear acceleration of the two phones **(b)** The LA_3a_ and the average value of LA3a of the two phones with 2 different orientations in rear trousers pocket.

In another aspect, based on the observation for a large number of people, we find that people usually put the mobile phone into the coat pocket vertically, which can result in four possible orientations: head upward and face inward, head upward and face outward, head downward and face inward, head downward and face outward. Three axis linear acceleration curves from two different smartphones worn in different orientations but the same rear trousers are presented in Figure 9(a). The curves of X, Y, Z axis linear acceleration from two smartphones are quite different due to the different orientations. However, while the acceleration magnitude which can measure the quantity of acceleration but with no directions required is chosen, it is insensitive for the orientations of the smartphones. Therefore, as shown in Figure 9(b), there were 2 similar LA_3a_ curves from two different mobile phones worn in the same rear trousers pocket with 2 different orientations. From the plot we can conclude that the orientations of the two mobile phones have no influence on the extracted features that we used for classification. Therefore, the proposed activity recognition solution is with high robustness.

## Conclusions

In this study, we investigated the physical activity recognition issue based on built-in sensors of the smartphone. Different from previous studies in which the phone must be attached to the subject’s body with fixed orientation and location, our approach is more convenient as that orientation and position of smartphone can be varying no matter the material and style of the hosting pocket. In order to improve classification performance, we explored orientation-independent features extracted from magnitudes as well as three axis direction components for recognizing five typical physical activities. Two different smartphones used in our experiments indicated that the universality of classification model with several typical activities recognized with good accuracy with the extracted statistical features. Overall, the approach we proposed is more feasible for long-term activity monitoring because of its high convenience, low cost, and the ability to classify several typical activities in daily life with a relatively high accuracy.

As the dataset is with large dimensional features and relatively small size of data, cross validation was used to assess the classification performance. However, due to the difference in way of performing activities around different subjects, the performance maybe varying in recognizing activities for data from external subjects with just seven subjects employed for training the classifier. In the future, we will recruit a large number of subjects to eliminate the inter-variability between different subjects and then validate the proposed models in external validation datasets to improve the robustness of the proposed solution. In addition, with the rapid development of wearable sensors such as embedded sensors in smart watch and clothes, a new kind of human activity recognition solution would be studied to monitoring the user’s activity at any time and any place. We also plan to develop a sport management application that can merge the physical activity collected from different wearable sensors based on a private cloud platform [32], and thus calculate the user’s physical activity and help them formulate a daily exercise program. In addition, security of data [33,34] will be considered in our future system.
